# A Fungus Developed in the Human Saliva

**Published:** 1886-12

**Authors:** 


### A Fungus Developed in the Human Saliva.

M. Galippe made known the following facts at a recent meeting of the Paris Academy of Medicine. After having purified saliva by means of Pasteur's filter, M. Galippe observed, at the lower extremity of the filter, which was not in contact with the fluid, a fungus composed of tubes and spores of mycelium. Following the advice of Professor Cornu, M. Galippe cultivated this fungus in the cells of Van Tieghem, and observed that the fungus was neither an *Aspergillus* nor a *Penicillium.* This fungus, which had neither been described nor represented, belongs to the Moniliæ family. M. Galippe proposed to give it the name of *Moniliæ sputicola.* M. Charcot repeated these statements at the Academy of Sciences, in the name of M. Galippe.

